# News and Notes

**Published:** 1901-09

**Authors:** 


### *NEWS AND NOTES.*

---

THE *Atlanta Medical and Surgical Journal* and *Southern Medical Record* having been consolidated into the ATLANTA JOURNAL-RECORD OF MEDICINE, exchanges will please be sent only to the latter. We are getting many duplicates.

---

THE City Board of Health in New Orleans has started a crusade against mosquitoes.

---

A CIRCULAR has been issued by General Wood, Governor of Cuba, strictly prohibiting the sale at the Cuban post exchanges of beverages containing any percentage whatever of alcohol.

---

THE antispitting ordinance is said to be strictly enforced in a number of Southern cities, while in Northern cities, notably Pittsburg and Buffalo, they find it difficult to enforce ordinances.

---

DR. STEPHEN HARRIS of Carrolton, was married on August 18, to Miss Haygood of Atlanta. Dr. Harris is now practicing medicine in Carrolton after having spent sometime in the Philippines in the volunteer medical service.

---

THE thirteenth annual meeting of the Tri-State Medical Society of Alabama, Georgia and Tennessee, will be held at the Tulane, Nashville, Tenn., Tuesday, Wednesday and Thursday, October 8, 9 and 10, 1901. The attendance promises to be large and an unusually attractive program will be presented. The railroads will give reduced rates. Those intending to read papers should send titles to the Secretary, Dr. Frank Trester Smith, Chattanooga, Tenn.

A CHICAGO hypnotist, according to the *Journal of the American Medical Association*, who applied to the Mayor for a permit to bury a young man alive while in a hypnotic state, has been summoned to appear before the State Board of Health on the charge of falsely representing himself to be a physician. His name does not appear on the list of registered physicians.

---

AMERICAN MEDICINE is responsible for the following: A curious superstition begins to gain a large number of supporters in Berlin. Three "doctors" named Grau, Freiberg, and Herrmann, have established a reputation for eradicating disease from men and beasts by driving it into trees. The cures are effected at night on Tuesdays and Fridays of the week of full moon. A needle is stuck into the skin over the affected part of the body, and remains there until a few drops of blood appear. The blood is collected on a sheet of paper, and the paper is rolled in the shape of a pill. The "doctor" then goes in a wood alone carrying this pill, selects a tree, and inoculates the bark with the pill. The process of blood-letting and inoculation is repeated three times.

---

AN interesting feature of next meeting of the Tri-State Medical Society at Nashville, Tenn., Oct. 8, 9, 10, 1901, will be a series of papers on sociological subjects. Some eight or ten papers will be presented, a list of which may be found elsewhere in this journal. These papers will be of interest to all professional men, as they will deal with the dangers and diseases that threaten our social life as a community, State and Nation. Every physician should not only have a medical knowledge of these subjects but his duty urges the use of all legitimate honorable means to disseminate such knowledge as will tend to lessen suffering and disease. These discussions will tend to awaken thought and action which will result in benefit to humanity. Every physician is especially invited to attend the meeting, take part in discussion of the papers and report of the sociological committee.

A WASHINGTON press dispatch is responsible for the following: According to scientific investigations, directed from Washington, which have been going on for several months, there are about 275 cases of leprosy in the United States—that is to say, that many have been reported. Very probably the real number is nearer 1,000 than 275, but this latter number is the highest that can be fixed with certainty. For various reasons physicians who have cases of this disease, in many instances, fail to report them; but the reported number is sufficiently large to cause some alarm. Of the 275 located cases, seventy-four are in New Orleans, among the foreign population, chiefly the Italian. There are twenty-three in Minnesota, mostly among Scandinavians, in the rural settlements. There are fifteen cases in North Dakota, and two in South Dakota, among the same class of people. So far as has been ascertained there are none in Michigan nor in Indiana, but in the big cities, Chicago has five cases and New York six. Boston has none.

---

THE next annual meeting of the Mississippi Valley Medical Association, under the Presidency of Dr. A. H. Cordier, of Kansas City, bids fair to eclipse all previous ones in attendance as well as scientific merit, as the following preliminary program will show. Unusual railroad rates have been obtained for this meeting—a one-fare rate by way of Cleveland, which will enable those taking advantage of these rates to obtain an extension of tickets to October 8th for attendance upon the Buffalo Exposition. A one-and-a-third fare rate on the certificate plan will be in effect via Detroit, Sandusky, and Toledo, with extension of return limit for only three days after the meeting. Put-in-Bay is an ideal place of meeting, the Hotel Victory a magnificent meeting site. The address in Medicine will be made by Dr. Frank Billings, of Chicago; the address in Surgery by Dr. Reginald Sayre, of New York City. The Association is to be congratulated on the selection of these two orators, who will acquit themselves in a most scholarly manner. The annual banquet will be held on the evening of the first day, September 12th; on the second evening, an evening will be given up to the reading of several papers with stereopticon exhibits and demonstrations; the President's address and the annual orations being delivered on the three mornings of the meeting. The profession is cordially invited to attend this meeting. No title can be received after August 20th for publication on the final program.

---

### AN IMPORTANT LAW—HOUSE BILL NO. 320.

*An Act to prevent the substitution of any drug in filling physicians' prescriptions by druggists in the State.*

SECTION 1. Be it enacted by the General Assembly of the State of Tennessee, That it shall be unlawful for any corporation, firm or person, or any combination or association of corporations, firms or persons engaged in the business of buying, compounding and selling drugs and medicines to substitute any drug or medicine in lieu or instead of that given to the patient by the physician on the face of his prescription.

SEC. 2. Be it further enacted, That it shall be unlawful for any agent or employe of such person, firm or corporation engaged in the business of buying and selling drugs in this State to substitute any medicine for the specific medicine mentioned in the physician's prescription.

SEC. 3. Be it further enacted, That any person, firm or corporation violating the provisions of this Act, or aiding or abetting the violation of the same, shall be guilty of a misdemeanor, and upon conviction shall be fined not less than $25 nor more than $100 for each and every offense.

SEC. 4. Be it further enacted, That this Act take effect from and after its passage, the public welfare requiring it.

Approved April 3, 1901. BENTON McMILLIN, Governor.

E. B. WILSON, Speaker House of Representatives.

NEWTON H. WHITE, Speaker of the Senate.

A true copy, JOHN W. MORTON, Secretary of State.

THE Thirteenth annual meeting of the Tri-State Medical Society of Alabama, Georgia and Tennessee will be held at the Tulane, Nashville, Tuesday, Wednesday and Thursday, October 8, 9 and 10, 1901.

The railroads will give reduced rates on the certificate plan.

The following papers have already been promised:

President's Address, M. C. McGannon, Nashville, Tenn.

Some Points in the Diagnosis and Treatment of Heart Disease, J. B. Marvin, Louisville, Ky.

Tuberculosis, Y. L. Abernathy, Hill City, Tenn.

Night Sweats of Phthisis, L. P. Barbout, Boulder, Colo.

Malaria as a Cause of Puerperal Eclampsia, E. O. Williamson, Gurley, Ala.

Static Electricity, J. B. Goodwin, Kingston, Tenn.

Smallpox, Wm. A. Duncan, Chattanooga, Tenn.

Remarks on Some Recent Cases, A. B. Ramsey, McMinnville, Tenn.

Anesthesia in General Practice, J. B., Murfreesboro, Tenn.

Anesthesia, German Haymore, Chattanooga, Tenn.

Local Anesthesia, Cooper Holtzclaw, Chattanooga, Tenn.

Local Anesthetics as Applied to Mucous Membranes, B. F. Travis, Chattanooga, Tenn.

Studies of the Blood and Urine in Connection with Anesthesia, H. Berlin, Chattanooga, Tenn.

Autoinfection, A. W. Boyd, Chattanooga, Tenn.

Migraine, Curran Pope, Louisville, Ky.

Report of Committee on Sociology, R. R. Kime, Atlanta, Ga.

a. Heredity and Acquired Characteristics as a Social Question.

b. Legislation and Its Limitation in Prevention of Crime and Disease, Hon. John Bell, Keeble, Nashville, Tenn.

c. Marriage and Heredity in Relation to Crime and Insanity, T. O. Powell, Milledgeville, Ga.

d. Tuberculosis; Its Prevalence and Prevention, J. D. Cromer, Atlanta, Ga.

e. Suppression of Consumption, R. C. Bankston, Birmingham, Ala.

f. Syphilis and Its Prevention as a Social Question, Childs & Champion, Atlanta, Ga.

g. Syphilis and Its Relation to Eye Disease, A. W. Sterling, Atlanta, Ga.

h. The Prevalence of Gonorrhea in the Male as a Social Question, W. Frank Glenn, Nashville, Tenn.

i. Gonorrhea in the Female with Suggestions as to Means of Prevention, W. G. Begart, Chattanooga, Tenn.

j. The Development and Control of the Sexual Instinct, J. W. Macquillan, Chattanooga, Tenn.

Softening of the Brain, Michael Campbell, Knoxville, Tenn.

Diagnosis and Treatment of Typhoid Fever, J. C. LeGrand, Birmingham, Ala.

A Plea for the Better Treatment of Physical Diagnosis in Southern Medical Colleges, Hazle Padgett, Columbia, Tenn.

Acute Dysentery; Some Experiences and Report of Cases, Allen E. Cox, Milan, Tenn.

Limitation in Abdominal Surgery, T. J. Crofford, Memphis, Tenn.

Conservative Treatment in Appendicitis, W. E. Fitch, Savannah, Ga.

Trachoma of the Female Genital Tract—A Plea for Its Early Recognition, J. A. Hale, Alto Pass, Ill.

Flat Foot, Michael Hoke, Atlanta, Ga.

Anatomy of the Genital Organs with Specimen, J. S. B. Woolford, Chattanooga, Tenn.

Gonorrhea, J. W. Johnson, Chattanooga, Tenn.

Diseases of the Sigmoid Flexure, W. L. Nelen, Chattanooga, Tenn.

Infant Feeding, C. S. Durand, Chattanooga, Tenn.

Scarletina and Its Complications, H. B. Wilson, St. Elmo, Tenn.

Brain Surgery; Report of Cases, Edwin B. Anderson, Chattanooga, Tenn.

Rheumatism; Cause or Effect in Affection of the Throat, W. Cheatham, Louisville, Ky.

The Work of the Laryngologist in Its Relationship to that of the General Practitioner, Richmond McKinney, Memphis, Tenn.

Superficial Foreign Bodies in the Eye, A. C. Corr, East St. Louis, Ill.

The Future of the Negro from the Standpoint of the Southern Physician, Seale Harris, Union Springs, Ala.

Title to be announced, W. D. Gaines, Lafayette, Ala.

Title to be announced, W. R. Blue, Louisville, Ky.

Title to be announced, Joseph Price, Philadelphia, Pa.

The Sanitation of Havana, Chas M. Blackford, Washington, D. C.

